# Early recurrence and ongoing parietal driving during elementary visual processing

**DOI:** 10.1038/srep18733

**Published:** 2015-12-22

**Authors:** Gijs Plomp, Alexis Hervais-Adelman, Laura Astolfi, Christoph M. Michel

**Affiliations:** 1 Functional Brain Mapping Lab, Department of Fundamental Neuroscience, University of Geneva, Geneva, Switzerland; 2 Perceptual Networks Group, Department of Psychology, University of Fribourg, Fribourg, Switzerland; 3 Brain and Language Lab, Department of Clinical Neuroscience, University of Geneva, Geneva, Switzerland; 4 Department of Computer, Control, and Management Engineering, University of Rome “Sapienza”, Italy

## Abstract

Visual stimuli quickly activate a broad network of brain areas that often show reciprocal structural connections between them. Activity at short latencies (<100 ms) is thought to represent a feed-forward activation of widespread cortical areas, but fast activation combined with reciprocal connectivity between areas in principle allows for two-way, recurrent interactions to occur at short latencies after stimulus onset. Here we combined EEG source-imaging and Granger-causal modeling with high temporal resolution to investigate whether recurrent and top-down interactions between visual and attentional brain areas can be identified and distinguished at short latencies in humans. We investigated the directed interactions between widespread occipital, parietal and frontal areas that we localized within participants using fMRI. The connectivity results showed two-way interactions between area MT and V1 already at short latencies. In addition, the results suggested a large role for lateral parietal cortex in coordinating visual activity that may be understood as an ongoing top-down allocation of attentional resources. Our results support the notion that indirect pathways allow early, evoked driving from MT to V1 to highlight spatial locations of motion transients, while influence from parietal areas is continuously exerted around stimulus onset, presumably reflecting task-related attentional processes.

Visual cortex consists of numerous hierarchically organized areas that form a densely connected network. Anatomical projections between these areas are often reciprocal: when a lower-level area projects to a higher-level one, a projection back to the lower-level area often exists^1^,^2^. Such reciprocal connectivity allows not only for bottom-up influence from lower- to higher-level areas in visual processing, but also for fast feedback from higher to lower ones.

Quickly after stimulus onset, visual activity propagates from primary visual cortex (V1) to widespread occipital, parietal and frontal areas, like the frontal eye fields (FEF)^3^,^4^,^5^. The first evoked activity recorded with EEG at the scalp is the C1 component, starting from around 50 ms after stimulus onset and peaking between 80 and 90 ms^6^. The C1 component reflects V1 activity but also activity in widely distributed regions situated in the occipital, parietal and frontal lobes^5^,^7^. Stimulus-evoked activity at latencies before 100 ms is traditionally considered a bottom-up process^8^. Even at these short latencies, however, there is mounting evidence of fast recurrent interactions between visual areas, obtained from direct recordings of neural activity in animal models^9^,^10^,^11^,^12^.

In addition to recurrent processing between visual areas, the parietal cortex and FEF are known to exert top-down influences that help flexibly adapt behavior to changing task demands and context^10^,^13^,^14^,^15^. Top-down interactions reflect task-specific processing of stimuli which is thought to typically arise at longer latencies after stimulus onset. At latencies between 100 and 200 ms more complex object processing occurs in the lateral occipital complex (LOC) which involves both recurrent processes and top-down interactions^16^,^17^,^18^,^19^. In surface EEG, this object processing is reflected by a posterior negativity, the N1 component.

Effects of top-down influence on visual processing are well-documented by experiments in which participants view identical stimuli under changing task demands. Activity in motion- and color-sensitive areas like MT and V4 depends on whether participants attend to stimulus motion or color, at latencies as early as 100 ms after stimulus onset^20^,^21^,^22^. Paying attention to motion increases activity throughout a network of areas involved in motion processing^23^. The effects of such top-down influence are not restricted to higher-level visual areas, attention can also affect activity in lower-level areas, including V1 around the C1 latency^24^,^25^,^26^. Even prior to stimulus onset a network of frontal and parietal areas is involved in switching between task demands^27^.

Top-down influence has thus been well studied by manipulating task-demands, but it is less clear what directed interactions from higher level areas take place in the absence of explicit, stimulus-related task demands in early visual processing. Here we use EEG and electrical source imaging to investigate the dynamics of recurrent interactions between visual areas and of top-down influence from attentional areas in human observers. We presented task-irrelevant checkerboards in the lower left or right visual field, which elicit well-characterized activation patterns in the EEG that can be accurately localized using EEG source imaging^5^,^7^,^28^.

To investigate the dynamics of two-way interactions in visual processing, directed connectivity measures with high temporal resolution are needed. Granger-causal modeling is an established statistical method to estimate the strength of directed interactions between brain areas^29^,^30^. Granger-causal methods quantify how well activity in one measured signal predicts future activity in the other measured signals. These methods can correctly identify the directed connectivity in model data and synthetic EEG signals^31^,^32^,^33^,^34^ and has found ample application in EEG recorded from humans^35^,^36^,^37^,^38^. We have recently validated a Granger-causal modeling approach based on multivariate autoregressive modeling that has high temporal resolution, spectrally weighted Partial Directed Coherence: (wPDC)^39^.

We hypothesized that by combining EEG source imaging and Granger-causal modeling in human observers, feedback and top-down influence can be identified and distinguished at short latencies. We first localized a network of 12 ROIs in each observer that included areas in occipital, parietal and frontal cortex for each observer using fMRI. We then applied wPDC to time-series of estimated current densities obtained from separate EEG measurements to identify the major drivers at the C1 and N1 peak latencies and determine whether they selectively targeted other areas. The results showed the expected bottom-up driving in the first 100 ms (i.e. from V1 to higher-level areas like MT and LOC), but in addition, we found fast recurrent interactions between MT and V1 in response to stimulation and a large, ongoing influence from parietal cortex already around stimulus onset.

## Results

### EEG results

**Figure 1:**
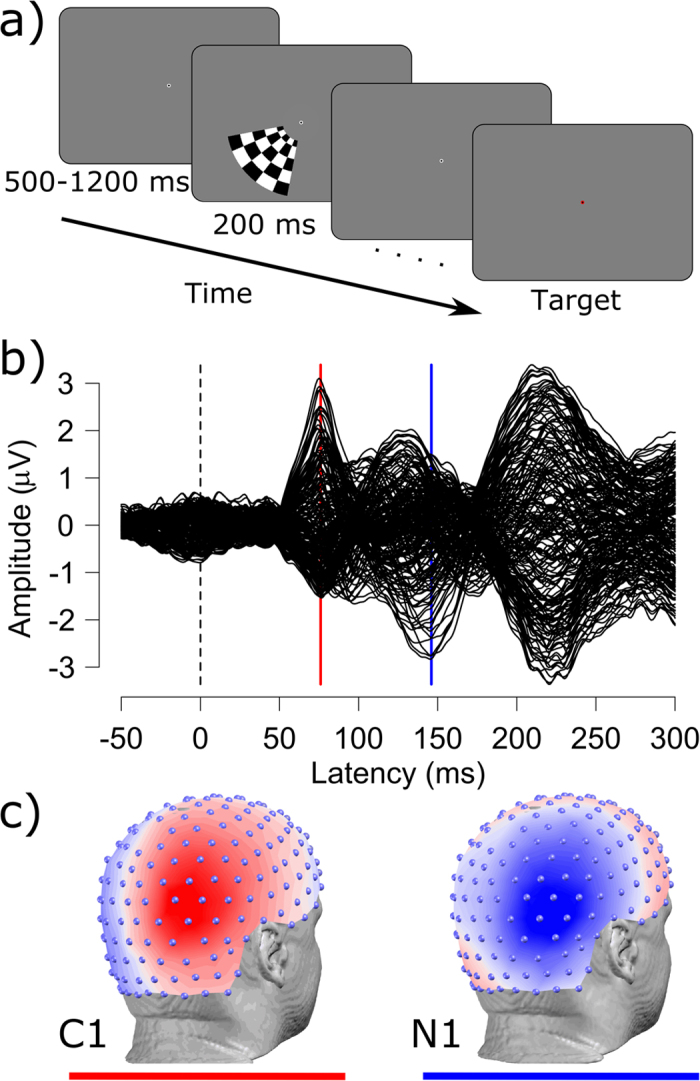
Paradigm and evoked response. (**a**) illustrates the stimulus presentation sequence. Checkerboards were presented for 200 ms in the lower left or right visual field while the task for participants was to maintain fixation on the center of the screen and respond when the fixation point changed to red (target). (**b**) shows the grand-average visual evoked response potential across 13 subjects. Each line reflects the average amplitude of one of the 204 electrodes used for analysis. (**c**) shows the topographic distributions across the scalp of visual evoked potentials at the C1 (76 ms) and N1 (146 ms) peak latency. Grand-average data projected onto the co-registered electrode positions of one participant.

We briefly presented checkerboard stimuli in the lower left or right visual field while participants detected a color change at the center of the screen. Visual evoked potentials on the scalp time-locked to checkerboard onset showed typical C1 and N1 components over the hemisphere contralateral to checkerboard presentation, peaking at 76 and 146 ms after stimulus onset (Fig. 1).

**Figure 2:**
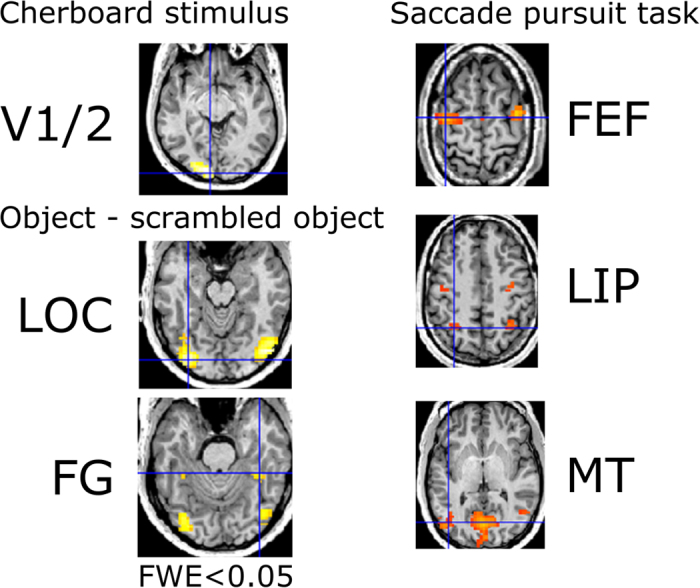
ROI localization in a representative individual participant, determined using three functional localizers. The blue cross indicates the location of the minimal p-value of the statistical contrast indicated. Source-space activity was estimated at these loci using EEG recorded in a separate session. See Methods for further details.

Twelve ROIs were successfully identified for each individual participant using data recorded in a separate fMRI session. The ROI locations are illustrated in Fig. 2 and their coordinates in MNI space are listed in Table 1.

To obtain activity estimates for each ROI from the EEG signal we used a distributed linear inverse solution (WMN) based on individual headmodels, thus taking into account each participant’s unique anatomy in the forward model (for further details, see Methods below)^40^,^41^. To determine the frequencies at which stimulus-specific processing was maximally present we subtracted for each ROI the normalized Power Spectral Density (PSD) response to checkerboards presented in the ipsilateral visual field from the PSD response to contralateral checkerboards.

**Figure 3:**
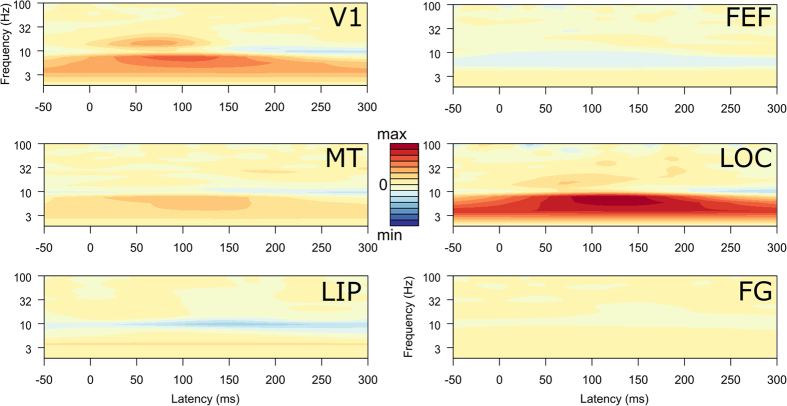
Stimulus-specific processing in the time-frequency domain. Time-frequency plots of the difference in evoked response to contra- and ipsilateral stimuli for each ROI (Fig. 2), based on EEG source-imaging (see Methods and text). V1 showed increased responses for contralateral stimulation in the beta and alpha band and LOC showed increased responses in the low alpha band. Scales are identical across plots.

The C1 component is to a large extent generated by activity in lower-level visual areas of the contralateral hemisphere^5^,^6^,^7^. PSD analysis of V1/V2 at the C1 peak latency (76 ms) showed more activity for contra- than ipsilateral stimuli in the alpha (8–13 Hz, 95% CI 0.01–0.09, *d* = 0.13) and beta band (13–30 Hz; 95% CI 0.01–0.06, *d* = 0.24), see Fig. 3. We focused the subsequent connectivity analysis at the C1 latency on the beta band because of the larger effect, compared to the alpha band. Furthermore, the beta band effect peaked around the C1 latency (Fig. 3), whereas the alpha band effect peaked at longer latencies. The N1 component is typically generated by extra-striate areas, including the LOC^5^,^42^. PSD analysis of LOC at the N1 peak latency (146 ms) showed increased activity in the alpha band only (95% CI 0.01–0.11, *d* = 0.24).

### Functional connectivity at the C1 latency, beta band

**Figure 4:**
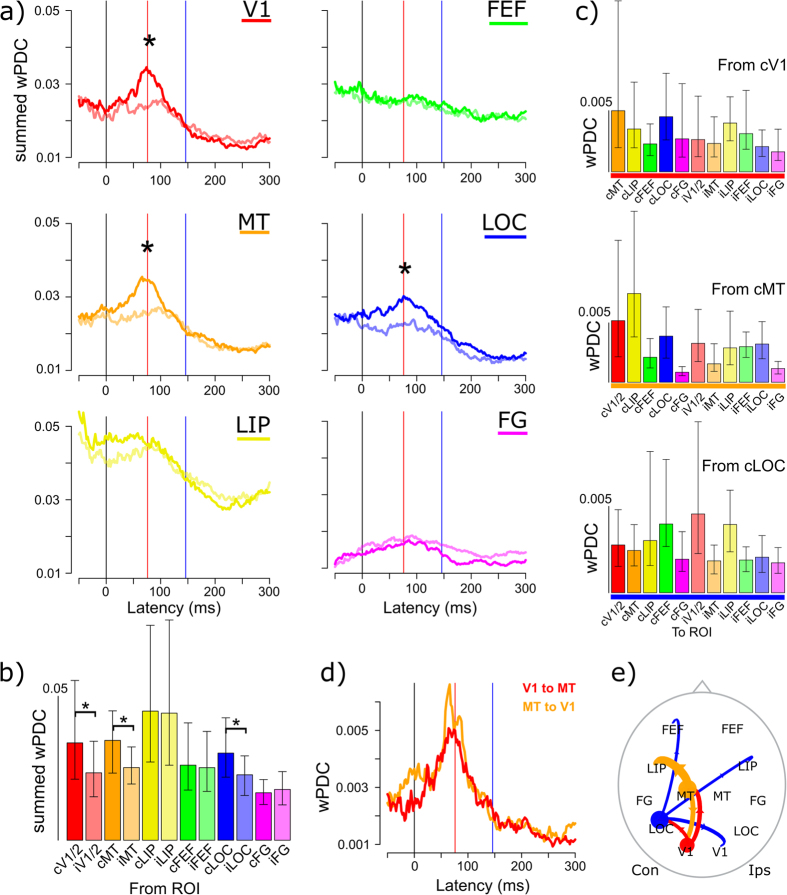
(**a**) Dynamics of summed driving from each ROI in the beta band, for stimuli in the contralateral and ipsilateral (semi-transparent colors) visual field. The red line indicates the peak latency of the C1 component, the blue one the peak latency of the N1 component. (**b**) shows the summed driving (wPDC) per ROI at the C1 peak latency (76 ms). (**c**) depicts driving from the stimulus-selective ROIs cV1, cMT and cLOC toward all other ROIs at the C1 peak latency. The color-codings are the same as those in (**a**). Error bars denote bootstrapped 95% confidence intervals around the mean, asymmetrically computed. (**d**) shows the functional interactions between V1 and MT after stimulus onset; the reciprocal nature of the interaction is reflected by the matching dynamics. (**e**) Shows a schematic top-view of the 12 individually localized brain areas (ROIs). Circle sizes reflect the relative summed driving per area, and arrow widths reflect relative driving strength at the C1 peak latency.

To analyze the total influence from each ROI we summed its outgoing functional connectivity strengths (wPDC) to all other ROIs. This summed driving reflects the total influence of the ROI on the network at a given time and frequency. At the C1 peak latency, V1, MT and LOC showed larger summed driving in the beta band for contralateral than ipsilateral stimulation. This stimulus effect selectively occurred around the peak latency of summed driving from these areas, coinciding with the peak latency of the C1 component (Fig. 4a,b).

Overall, lateral intraparietal cortex (LIP) in both hemispheres showed the largest summed driving at the C1 peak latency (Fig. 4b,c), and this driving was similar for contra- and ipsilateral stimuli.

We next analyzed whether the areas showing a stimulus effect showed specific driving toward other ROIs. We found that the main targets of contralateral V1 (cV1) were cLOC and cMT, in line with a feed-forward information flow from V1 toward higher-level visual areas (Fig. 4c). The main targets of cMT were cLIP and cV1, while cLOC specifically targeted cFEF, but also iLIP and iV1. The influence from MT and LOC back to V1 indicates that feedback to primary areas already occurs at short latencies. The dynamics of the two-way cV1-cMT interaction is plotted in Fig. 4d, showing that both peak just before the peak of the C1 component.

### Functional connectivity at the N1 latency, alpha band

**Figure 5:**
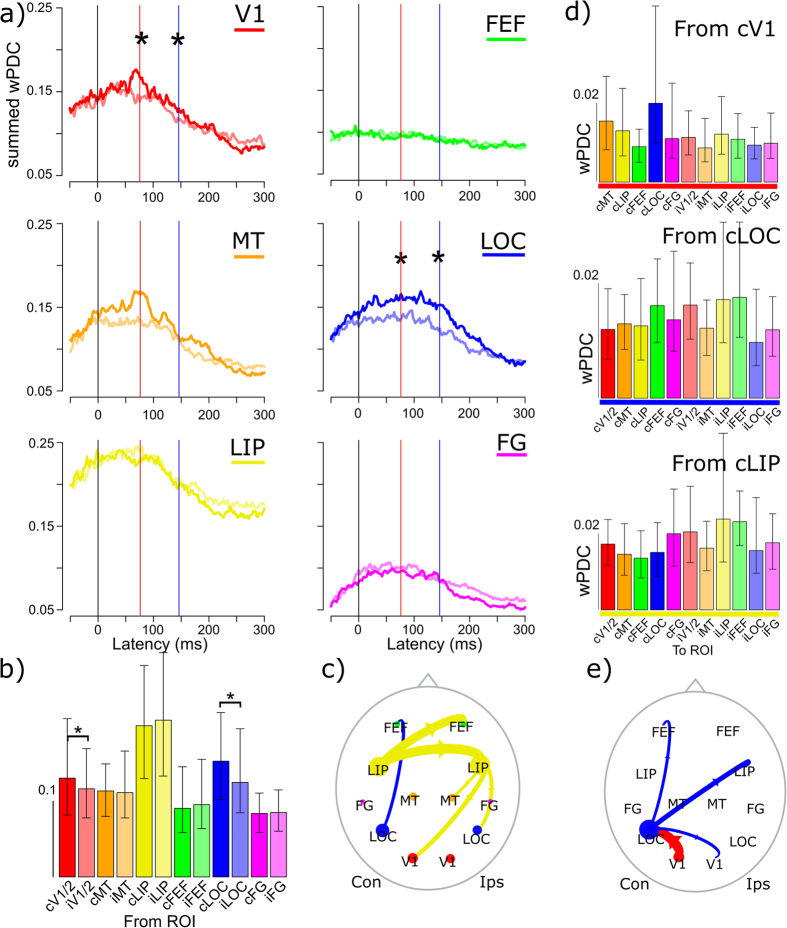
(**a**) Dynamics of summed driving (wPDC) from each ROI in the alpha band, for stimuli in the contralateral and ipsilateral (semi-transparent colors) visual field. Identical color-coding is used for the barplots; significant differences are indicated with an asterisk, see Methods. (**b**) Shows the summed driving at the N1 peak latency for each ROI. Panel (**c**) illustrates the 5% strongest connections at this latency. (**d**) Shows the driving from cV1, cLOC and cLIP to the other ROIs. (**e**) summarizes the directed connectivity from stimulus selective ROIs at the C1 peak latency. Plotting conventions are the same as those in Fig. 4.

At the N1 peak latency stimulus effects were most pronounced in the alpha band (Fig. 3). Granger-causal modeling results in the alpha band showed significantly larger driving from LOC and V1 for contralateral than ipsilateral stimuli (Fig. 5a,b) at this latency. Overall, bilateral LIP showed the largest summed driving at this latency. The LIP driving exceeded the driving from MT or FEF and did not show a stimulus effect. Figure 5c illustrates the 5% strongest directed connections at the N1 latency, suggesting a dominant influence from LIP in visual processing around the N1 latency.

The main directions of influence from cV1, cLOC and cLIP are shown in Fig. 5d. The main target of cV1 was cLOC while the main targets of cLOC were contra-and ipsilateral FEF, iLIP and iV1. This connectivity from stimulus-specific areas is summarized in Fig. 5e. The main targets of cLIP were cV1 and iFEF. Large driving to iLIP was also seen, in line with known structural connectivity between homologues.

## Discussion

To study the dynamics of recurrent interactions and top-down influence in early visual processing of human observers we combined EEG source imaging and Granger-causal modeling in a network of twelve ROIs that included visual and attentional areas in occipital, parietal and frontal cortex. The directed functional connectivity results at the C1 peak latency showed a pattern of driving largely in line with feed-forward information flows from V1 and MT. However, recurrent driving from MT toward V1 was also observed at short latencies. In addition, the results suggest that LIP is an important network driver at both the C1 and N1 peak latencies, particularly in the alpha band.

Previous studies using directed functional connectivity measures have shown evidence for top-down interactions in visual processing. Early PET and fMRI studies used structural equation modeling to show a role for directed interactions from lateral frontal areas in visual processing^43^,^44^. Simultaneous recordings from area MT and parietal areas in monkeys has shown that activity increases in parietal areas precede those in MT during top-down attention, suggesting that parietal areas drive the activity increases in MT^45^. Top-down influence from parietal cortex and FEF onto V4 was demonstrated in a spatial attention task with fMRI and Granger-causal modeling^14^. The directed connectivity between areas in the dorsal attention network has been shown to correlate with response times^46^. Granger-causal modeling has been applied to investigate object processing using limited number of ROIs and a non-time-varying approach across the evoked response^37^. This work showed two-way interactions between infero-temporal, parietal and frontal areas in object processing.

These past studies, however, have been unable to provide a dynamic characterization of top-down influence because they used fMRI or PET recordings that have insufficient temporal resolution, or analyzed interactions across time. Here, we used Granger-causal modeling with high temporal resolution to investigate two-way interactions in visual processing.

At the C1 latency, stimulus effects in the PSD were largest in the beta band, whereas at the N1 latency, stimulus effects appeared in the lower alpha band (Fig. 3). Activity in the alpha band is associated with thalamo-cortical loops and top-down interactions reflecting attention^47^,^48^. The functional significance of beta band activity in vision is less well understood, but is thought to reflect both local cortical activity and cortical feedback in large-scale networks^48^,^49^,^50^,^51^. Although functional differences between frequency bands were not the focus of our investigation, and we selected frequency bands for analysis in a data-driven way, our results are broadly in line with recent proposals that top-down processing may occur at lower frequencies while feedback and recurrent processing occurs at higher frequencies^51^,^52^. In line with this, the driving from LIP was most pronounced in the alpha band and showed no bottom-up stimulus effects, while stimulus effects and recurrent processing were most pronounced in the beta band. Nevertheless, our data also showed stimulus-specific effects in the alpha band, even in V1/2 where there can only be feedforward driving. In addition, area MT exerted simultaneous bottom-up influence to LIP and top-down influence to V1 in the beta band, suggesting that in our data at least, frequency cannot be equated with function.

Connectivity results around the C1 latency showed larger summed driving from V1, MT and LOC for contralateral stimuli. MT is activated quickly after stimulation, in line with known bottom-up spread of activity in the contralateral hemisphere. Activity in LOC can also occur at short latencies^3^,^8^,^53^. It is therefore plausible that these areas exert more influence after contra- than ipsilateral stimuli already at short latencies. At the N1 latency, driving from MT no longer showed a stimulus effect, indicating that stimulus-specific driving from MT is confined to earlier latencies, in line with its fast response properties. In contrast, LOC and V1 continue to exert more influence for contra than ipsilateral stimuli at longer latencies, in line with their greater role in processing visual content.

The main targets of cV1 at the C1 latency were cLOC and cMT, in line with a feed-forward spread of activity from V1 toward higher-level visual areas (Fig. 4c,e). At this latency cMT predominantly targeted cLIP, in agreement with known structural and functional connectivity of the dorsal attentional network, which aids fast visual processing of spatial properties and motion^3^,^13^.

The results also showed a fast two-way interaction between cV1 and cMT around the C1 peak latency (76 ms after stimulus onset). Direct interactions between MT and V1 are possible through monosynaptic, reciprocal projections^1^,^12^,^54^. The current results indicate that V1 activity is already co-determined by MT activity at short latencies after stimulus onset, suggesting that the fast propagation of activity from V1 to MT is not a uniquely bottom-up process but includes feedback from MT onto V1. The coupling between V1 and MT peaked around the C1 latency and diminished afterwards (Fig. 4d). The results are in line with a previous study showing top-down influence from MT to V1 at latencies before 100 ms^55^. This work used motion stimuli to show that MT top-down influence was specific for low-contrast moving stimuli, it did not occur for high-contrast stimuli.

How could driving from MT to V1 arise at these short latencies? In primates, initial MT activation can co-occur with the initial activation of V1^3^,^53^, through two distinct mechanisms. First, through fast magnocellular connections from V1 to MT^12^,^54^. Or alternatively through the direct koniocellular pathway from the LGN to MT, a pathway that enables rapid detection of motion^56^,^57^,^58^. In our results driving from MT to V1 occurs at similar latencies and without being systematically delayed with respect to the V1 to MT driving. This favors the interpretation that a route bypassing V1 allows MT to directly influence afferent activity in V1.

Evidence for the importance of fast MT–V1 interactions in human comes from a TMS study that showed that interference with V1 can abolish phosphene percepts elicited by TMS over MT, but only when TMS over V1 follows the TMS over MT at short latencies^59^. What could be the function of the early influence from MT? In animal models, feedback from MT has been shown to increase the responsiveness of V1 neurons, particularly for small, low-salience, signals^60^,^61^. Early recurrent processing may thus serve to disambiguate suboptimal visual input^10^,^62^. MT provides a fast, but coarse interpretation of the scene or the changes therein, a coarse scene analysis is fed back from MT to V1, the M signal that informs the later P-signal^63^. In our data the MT driving may help process the transients caused by stimulus onset.

We found a second instance of feedback influence around the C1 latency in the driving from cLOC toward iV1 (Fig. 4). This driving toward lower level-areas in the other hemisphere is in line with callosal projections from V1 to extrastriate areas including V4 have been shown in monkey^64^, under the assumption that these connections are reciprocal, possibly relating information about global stimulus content.

The fast interactions described here support the view that even simple, task-irrelevant visual stimuli evoke fast, two-way interactions between multiple areas^9^,^10^,^54^,^62^. Reciprocal interactions among visual areas at short latencies may help to disambiguate visual objects, both in terms of their spatial location or motion, and in terms of their content^10^. Our stimuli were clearly visible checkerboards occurring at unpredictable intervals. The early driving from MT may therefore inform V1 of where in the visual field a change has happened. Whether this MT driving of V1 depends on disambiguation effort should be tested in future experiments that vary the visibility of the stimuli or the uncertainty about their content.

In addition to specific recurrent interactions between visual areas, our findings also demonstrate interactions involving LIP and FEF, whose activity is known to reflect attentional processes^13^,^14^,^15^. We found that targets of LOC driving involve LIP and FEF, at both the C1 and N1 latencies (Figs 4c,5d), including some in the ipsilateral hemisphere. These functional connections are in line with known interactions between the dorsal and ventral visual streams^54^. We could speculate that this effect may be due to the nature of our stimulus paradigm. To ensure attention to the center of the screen and reduce eye-movements, participants were instructed to maintain fixation on a central dot and report its color change while checkerboards were presented in the periphery. The central fixation task was designed to minimize top-down processes like expectations about and attention to the content of the checkerboard stimuli. That LOC predominantly relayed information to areas associated with attention could possibly reflect processing of the spatial location of the stimuli, which were randomly presented in the left or right visual field. Had the task demands required a detailed inspection or disambiguation of visual content we would have expected LOC to interact more with other visual areas^10^,^54^.

The results identified surprisingly strong and ongoing driving from LIP, already at stimulus onset. LIP is a key area of the dorsal attention network for top-down control of visual attention^13^. Although we did not directly manipulate task-demands, successfully detecting color changes at the center of the screen requires sustained attentional processes. Given that attention had to be maintained, and the known role of LIP in top-down allocation of attention, a likely interpretation of the strong and persistent driving from LIP is that it reflects task-related top-down influence. This interpretation is in line with a previous connectivity study that showed increased pre-stimulus connectivity between parietal, frontal and occipital areas in anticipation of a difficult task^27^, and with a Granger-causal modeling study showing the role of parietal cortex in top-down control^14^.

In addition, LIP driving did not change with stimulus location and was strongest in the alpha band. LIP plays a role in spatial attention which are known to manifest in the alpha band^47^. During focal attention, increased alpha-band power has been shown to suppress activity in unattended locations^65^. The driving from LIP may thus function to suppress activity in the receptive fields of the bilateral stimulus locations while participants maintained central fixation.

Our results, LIP appears to modulate activity in various, broadly distributed areas, in line with its widespread structural connectivity^66^. Although the largest influence from LIP targeted ipsilateral LIP and FEF, the targets of LIP driving were less specific than those from e.g. V1 (Fig. 5d). This suggests that LIP orchestrates spatial attention by modulating activity throughout the network, and not by specific targeting of a limited number of areas, at least under the current task conditions.

The dominance of LIP over time (Fig. 5a) suggests that LIP driving is an ongoing process that plays an important part in shaping the evoked response. In our data driving from lower-level areas never exceeded that from LIP. This suggests that LIP driving plays an important part in the evoked response, and that the total influence of LIP can exceed the influence from early visual areas in the processing of simple stimuli. We note that our interpretation of LIP driving as a task-related top-down process is a tentative one; further experiments that directly manipulate the strength of top-down interactions under changing task conditions should help further clarify the targets of LIP driving and its timing.

In sum, our directed connectivity results distinguished a pattern of fast feed-forward driving in visual processes that co-exists with top-down driving from MT onto V1 at short latencies. LIP appears to play a considerable role in evoked visual responses through ongoing driving that most-likely reflects task-related processes. Our results suggest that vision is best viewed as a fast, highly interactive processes distributed among cortical areas. That we were able to separate fast feedback and top-down attentional processing in human by combining EEG source imaging and Granger-causal modeling is a promising basis for future studies in which stimulus and tasks properties are independently varied to better understand the functional roles of feedforward, recurrent and top-down processing.

## Methods

### Participants

Sixteen right-handed participants took part in the experiment (mean age 31, range 20–33 years; 5 female) with good visual acuity (mean 1.5, range 0.8–1.7), as measured by the Freiburg visual acuity test^67^. All participants gave informed consent before the experiment. All experimental methods and procedures complied with the Declaration of Helsinki and were approved by the ethics committee of the University of Geneva.

### Stimuli and apparatus

The checkerboard stimulus was a circular wedge (70 degrees of arc), offset by 2 degrees from the center of the screen and subtending 6 degrees of visual angle. Polarity changes (black/white) where spaced 3 cycles per degree radially, and 12 cycles per degree angularly (Fig. 1).

Object images came from the publicly available SVLO database^68^ and subtended about 3 degrees of visual angle. Luminance-matched control stimuli were created by fully randomizing the phases in the Fourier domain for each image^69^.

The same stimuli were used in the fMRI and the EEG session. Stimuli were back-projected onto a screen during fMRI recordings, and presented on a CRT monitor during EEG recordings. Stimulus generation, presentation and timing was controlled with PsychoPy software^70^,^71^ run under Python 2.7. Correct stimulus onset timing and duration were verified using a photo-diode.

### fMRI data acquisition and pre-processing

We used MRI to acquire structural brain images and to functionally localize bilateral regions of interest (ROIs) that respond to different aspects of visual processing, namely primary visual areas (V1/2), lateral occipital cortex (LOC), frontal eye fields (FEF), lateral intraparietal area (LIP) and the middle temporal motion area (MT).

Participants were scanned on a whole-body Tim Trio system (3T; Siemens Healthcare), at the Brain and Behaviour Laboratory at the University of Geneva, with a radio-frequency (RF) body transmitter and a 32-channel receiver head coil. Each scanning session consisted of an initial localizer scan, enabling appropriate centering of the field of view for subsequent scans. Three T2*-weighted echo-planar-imaging (EPI) functional runs (axial orientation, 3*3 mm in-plane resolution, 3.2 mm slice-thickness, TR = 2100 ms, TE = 30 ms, angle of flip = 80°) were acquired, followed by a T1-weighted structural image (sagittal orientation, 1 mm isotropic voxels, TR = 1900 ms, TE = 2.27 ms, TI = 900 ms, FoV = 256*256). The three functional localizer runs are described below.

In the localizer run for V1/2, participants were required to fixate a central dot and to indicate with a button-press (using their right index finger) when they detected a change in its color. A block design was employed in which checkerboard stimuli were presented successively in the lower left and right visual field. Checkerboards remained on screen for between 15 and 23 seconds (7–11 TRs) while their contrast polarity was flipped at random intervals (100–300 ms). Every pair of left-then-right checkerboards was followed by a period of variable duration (13–21 s), during which only the fixation dot remained on screen. A total of fifteen blocks were presented, with a total duration of 4.5 min (130 TRs) that remained identical over participants.

To localize LOC we presented object and scrambled stimuli in the center of the screen for 300 ms, ISI varied randomly (200–500 ms). Participants were instructed to press a button when a target picture (a red flag) was presented. This decoy task was employed to keep participants alert and focused on the content of the images. Stimulus presentation was blocked with blocks lengths varying between 15 and 23 seconds (7–11 TRs), and each block of stimulation was followed by a period of variable duration (10–21 s) during which only the fixation dot remained on screen. A total of twenty blocks were presented, with a fixed total duration of 6.5 min (165 TRs).

FEF, LIP area MT were localized using a saccadic pursuit task^72^. A fixation dot remained in a fixed location for a random period (0.5–1 s) and then appeared at a random location within 6 degrees horizontally and 4 degrees vertically of the center of the screen. A block design was used in which pursuit blocks lasting between 15 and 23 seconds (7–11 TRs) alternated with periods of fixation lasting between 13 and 21 seconds (6–10 TRs). Five blocks of pursuit and five blocks of fixation were acquired, with a fixed total duration of 3 minutes (85 TRs).

Analysis and preprocessing was carried out using SPM 8, running in Matlab R2010b (The Mathworks Inc., Natick, MA., USA). Preprocessing for each subject included: 1) rigid realignment of each EPI volume to the first in the session, 2) co-registration of the structural volume to the mean EPI image, and 3) spatial smoothing using a Gaussian kernel of 8 mm.

Fixed-effects analyses were carried out separately for each localizer, using a general linear model for each participant in which every scan was coded for condition, and null events were left unmodeled. The analysis was carried out in the participants’ native space in order to permit comparability of the single-subject fMRI results with the single-subject EEG results. In order to allow identification of functional activations according to existing templates, and to make the reporting of results comparable with the existing literature, the normalization parameters warping each participant’s T1 scan to MNI template space were calculated.

V1/V2, MT, FEF and LIP were localized using statistical contrasts with baseline. LOC was localized by a statistical contrast between images of objects and phase-scrambled versions of those objects. This localizer also separately identified fusiform gyrus (FG) in each subject.

The 12 ROIs were successfully localized in each participant. Where possible, a statistical threshold of p < 0.05, corrected for familywise-error was applied. Otherwise, the peaks were identified at a threshold of p < 0.001 uncorrected (saccadic pursuit localizer, seven participants, one or two ROIs per participant) or p < 0.01 (object localizer, one participant, single ROI).

### EEG recording and processing

EEG was recorded on a separate day in an electrically shielded room with a 256 channel EGI Geodesic setup (EGI Eugene, OR, USA). Recordings were referenced against the Cz electrode, the impedances were kept below 50 kΩ, data were digitized at 1000 Hz.

Checkerboard patterns were presented for 200 ms in the lower left or right visual field (200 repetitions per location) with a random presentation order and randomly varying inter-stimulus intervals (500–1200 ms).

Participants sat at 1 m from the screen and were instructed to maintain fixation on a small disc (0.2 degree of visual angle) placed centrally on the screen during the entire recording block. Recording blocks lasted about 4 minutes, breaks were offered in between.

To help maintain attention on the center of the screen participants were instructed to press a button when the fixation point occasionally changed to red. Color changes of the fixation point were correctly detected in 93% of cases (range across participants 68–100%), showing good task-compliance. For three participants intermittent response box failures precluded the collection of behavioral data during EEG recordings. In those cases we verified task-compliance immediately after the recording block.

Individual electrode positions were digitized in 3D with a photogrammetry system (EGI Eugene, OR, USA). Off-line, recordings were DC-corrected, high-pass filtered at 0.1 Hz and divided into epochs between −100 and 400 ms around stimulus onset. We excluded the cheek and lower neck electrodes for the further analysis, keeping 204 electrodes. Epochs were visually inspected and those with muscle or eye-movement artifacts and/or amplitudes over 75 μV were excluded. On average 163 Left and 163 Right stimulation epochs per participant were kept for further analysis. Noisy channels were identified and interpolated using 3D spherical splines^73^. On average 7 out of 204 channels were interpolated per participant. We removed 50 Hz line noise using a multitaper^74^.

We derived current density time-series for each epoch using a distributed linear inverse solution (weighted minimum norm), implemented in the freely available Cartool software^40^. We co-registered the digitized 3D electrode layouts to each participant’s MRI and defined a solution space of about 5000 source points, evenly spaced within the participant’s grey matter. For each of the 12 ROIs we selected the source point closest to the minimum *p*-value, as obtained by the fMRI localizers. To calculate individual lead fields we used realistic, analytical head models constructed from individual MRIs using a manifold of locally adapted spheres^40^,^41^. Within ROIs, scalar current density values were obtained by projecting instantaneous 3D dipoles to the predominant evoked dipole direction in each ROI, determined between 50 and 400 ms after stimulus onset^75^,^76^.

Directed connectivity between the ROIs was estimated using Granger-causal modeling of z-scored single trial data in the 1–100 Hz range^29^,^30^,^32^. Specifically, we used time-varying MVAR estimation based on Kalman filtering^77^ and row-normalized, spectrally weighted Partial Directed Coherence (wPDC;^39^,^78^). Instantaneous power spectral density (PSD) in the 1–100 Hz range was calculated for each ROI by inverting complex decompositions (S-transform) on the scalp^28^,^79^ at the single epoch level. PSD and PDC values were normalized within participants across contra- and ipsilateral ROIs before scaling.

For each ROI we summed the outgoing wPDC strengths toward the other 11 ROIs as a measure of the total driving influence on the network.

### Statistical analyses

The analyses of the EEG and connectivity data aimed to limit the number of statistical tests by selecting a small number of ROIs from the more than 5000 possible source-points. In addition, we used a data-driven approach to restrict testing to the C1 and N1 peak latencies, as determined by the grand-average ERP (Fig. 1), and to three frequency bands critical to the evoked response, the alpha (8–13 Hz), beta (13–30 Hz) and gamma (30–60 Hz).

At the C1 and N1 latency we determined whether responses to contralateral stimuli were increased with respect to those of ipsilateral stimuli for each frequency band, using a non-parametric bootstrapping approach (n = 10000) to avoid unwarranted assumptions of normality. This analysis was carried out on normalized individual PSD responses, using the same data used for spectral scaling of the PDC. Subtracting ipsilateral from contralateral responses assures that the stimulus-driven response is isolated. Ipsilateral activity provides a good control because activity is not expected in the ipsilateral hemisphere until later on. It also controls against the limited spatial resolution (spatial leakage) of inverse solutions and provides a simple alternative to computationally intensive and competing surrogate data approaches^31^,^80^,^81^.

At the C1 and N1 peak latency we bootstrapped 95% confidence intervals (CI) of the difference between evoked responses contra- and ipsilateral to stimulation. If the lower bound of the 95% CI exceeds zero, the contralateral response was deemed to exceed that of the ipsilateral response, and the null hypothesis of equal amplitudes for contra- and ipsilateral stimuli rejected. We calculated effect sizes using Cohen’s *d* with pooled s.d.s in the denominator^82^.

In the thus selected frequency bands we determined whether the summed wPDC from each contralateral ROI exceeded that of the corresponding ipsilateral ROI by bootstrapping 95% CIs of the difference between their summed wPDC values.

For the ROIs that showed increased driving for contralateral stimuli we calculated the average wPDC to all other ROIs and their 95% CIs. This way we explored the strengths of driving toward the other ROIs and determined whether specific ROIs were targeted, thus refraining from arbitrary testing between wPDC strengths toward the 11 other ROIs and from comparing all possible connections in strength without theoretical motivation. The 95% confidence interval provides a useful estimate of the variability in the sample and the range in which the true values are most likely to fall^83^.

For connectivity plots, we adapted functions from the eConnectome toolbox^84^.

## Additional Information

**How to cite this article**: Plomp, G. *et al.* Early recurrence and ongoing parietal driving during elementary visual processing. *Sci. Rep.* **5**, 18733; doi: 10.1038/srep18733 (2015).

## Figures and Tables

**Table 1:** Average x, y, z coordinates of ROI locations in MNI space (mm).

ROI	x (s.d.)	y (s.d.)	z (s.d.)
L V1	−14 (5)	−100 (3)	7 (7)
R V1	17 (5)	−97 (3)	9 (4)
L MT	−48 (6)	−69 (8)	7 (6)
R MT	50 (7)	−66 (9)	11 (9)
L LIP	−31 (7)	−54 (7)	60 (4)
R LIP	30 (9)	−51 (10)	61 (4)
L FEF	−39 (7)	−7 (4)	56 (8)
R FEF	40 (10)	−4 (4)	55 (10)
L LOC	−49 (8)	−80 (7)	−5 (5)
R LOC	46 (4)	−76 (10)	−8 (8)
L FG	−40 (4)	−51 (6)	−18 (3)
R FG	43 (4)	−48 (10)	−17 (4)

## References

[b1] Felleman D. J. & Van Essen D. C. Distributed Hierarchical Processing in the Primate. Cereb. Cortex 1, 1–47 (1991). 1822724 10.1093/cercor/1.1.1-a

[b2] Markov N. T. *et al.* A Weighted and Directed Interareal Connectivity Matrix for Macaque. Cereb. Cortex 24, 17–36 (2014). 23010748 10.1093/cercor/bhs270 PMC3862262

[b3] Bullier J. Integrated model of visual processing. Brain Research Reviews 36, 96–107 (2001). 11690606 10.1016/s0165-0173(01)00085-6

[b4] Kirchner H., Barbeau E. J., Thorpe S. J., Régis J. & Liégeois-Chauvel C. Ultra-Rapid Sensory Responses in the Human Frontal Eye Field Region. J. Neurosci. 29, 7599–7606 (2009). 19515928 10.1523/JNEUROSCI.1233-09.2009 PMC6665413

[b5] Plomp G., Michel C. M. & Herzog M. H. Electrical source dynamics in three functional localizer paradigms. NeuroImage 53, 257–267 (2010). 20600987 10.1016/j.neuroimage.2010.06.037

[b6] Jeffreys D. A. & Axford J. G. Source locations of pattern-specific components of human visual evoked potentials. I. Component of striate cortical origin. Exp Brain Res. 16, 1–21 (1972). 4646539 10.1007/BF00233371

[b7] Di Russo F., Martínez A., Sereno M. I., Pitzalis S. & Hillyard S. A. Cortical sources of the early components of the visual evoked potential. Hum. Brain Mapp. 15, 95–111 (2002). 11835601 10.1002/hbm.10010 PMC6871868

[b8] Lamme V. A. F. & Roelfsema P. R. The distinct modes of vision offered by feedforward and recurrent processing. Trends in Neurosciences 23, 571–579 (2000). 11074267 10.1016/s0166-2236(00)01657-x

[b9] Chen M. *et al.* Incremental Integration of Global Contours through Interplay between Visual Cortical Areas. Neuron 82, 682–694 (2014). 24811385 10.1016/j.neuron.2014.03.023

[b10] Wyatte D., Jilk D. J. & O’Reilly R. C. Early recurrent feedback facilitates visual object recognition under challenging conditions. Front. Psychol 5, 674 (2014). 25071647 10.3389/fpsyg.2014.00674 PMC4077013

[b11] Sandell J. H. & Schiller P. H. Effect of cooling area 18 on striate cortex cells in the squirrel monkey. Journal of Neurophysiology 48, 38–48 (1982). 6288886 10.1152/jn.1982.48.1.38

[b12] Nassi J. J., Lyon D. C. & Callaway E. M. The Parvocellular LGN Provides a Robust Disynaptic Input to the Visual Motion Area MT. Neuron 50, 319–327 (2006). 16630841 10.1016/j.neuron.2006.03.019 PMC3398675

[b13] Corbetta M. & Shulman G. L. Control of goal-directed and stimulus-driven attention in the brain. Nat Rev Neurosci 3, 201–215 (2002). 11994752 10.1038/nrn755

[b14] Bressler S. L., Tang W., Sylvester C. M., Shulman G. L. & Corbetta M. Top-Down Control of Human Visual Cortex by Frontal and Parietal Cortex in Anticipatory Visual Spatial Attention. J. Neurosci. 28, 10056–10061 (2008). 18829963 10.1523/JNEUROSCI.1776-08.2008 PMC2583122

[b15] Gilbert C. D. & Li W. Top-down influences on visual processing. Nat Rev Neurosci 14, 350–363 (2013). 23595013 10.1038/nrn3476 PMC3864796

[b16] Chicherov V., Plomp G. & Herzog M. H. Neural correlates of visual crowding. NeuroImage 93, Part 1, 23–31 (2014). 24582921 10.1016/j.neuroimage.2014.02.021

[b17] Grill-Spector K., Kourtzi Z. & Kanwisher N. The lateral occipital complex and its role in object recognition. Vision Research 41, 1409–1422 (2001). 11322983 10.1016/s0042-6989(01)00073-6

[b18] Grill-Spector K. & Weiner K. S. The functional architecture of the ventral temporal cortex and its role in categorization. Nat Rev Neurosci 15, 536–548 (2014). 24962370 10.1038/nrn3747 PMC4143420

[b19] Shpaner M., Molholm S., Forde E. & Foxe J. J. Disambiguating the roles of area V1 and the lateral occipital complex (LOC) in contour integration. NeuroImage 69, 146–156 (2013). 23201366 10.1016/j.neuroimage.2012.11.023 PMC3872825

[b20] Chawla D., Rees G. & Friston K. J. The physiological basis of attentional modulation in extrastriate visual areas. Nat Neurosci 2, 671–676 (1999). 10404202 10.1038/10230

[b21] Schoenfeld M. A., Hopf J.-M., Merkel C., Heinze H.-J. & Hillyard S. A. Object-based attention involves the sequential activation of feature-specific cortical modules. Nat Neurosci 17, 619–624 (2014). 24561999 10.1038/nn.3656

[b22] Zhang W. & Luck S. J. Feature-based attention modulates feedforward visual processing. Nat Neurosci 12, 24–25 (2009). 19029890 10.1038/nn.2223

[b23] Büchel C. *et al.* The functional anatomy of attention to visual motion. A functional MRI study. Brain 121, 1281–1294 (1998). 9679780 10.1093/brain/121.7.1281

[b24] Lee T. S., Yang C. F., Romero R. D. & Mumford D. Neural activity in early visual cortex reflects behavioral experience and higher-order perceptual saliency. Nat Neurosci 5, 589–597 (2002). 12021764 10.1038/nn0602-860

[b25] Poghosyan V. & Ioannides A. A. Attention Modulates Earliest Responses in the Primary Auditory and Visual Cortices. Neuron 58, 802–813 (2008). 18549790 10.1016/j.neuron.2008.04.013

[b26] Rauss K. S., Pourtois G., Vuilleumier P. & Schwartz S. Attentional load modifies early activity in human primary visual cortex. Hum. Brain Mapp. 30, 1723–1733 (2009). 18711710 10.1002/hbm.20636 PMC6871007

[b27] Phillips J. M., Vinck M., Everling S. & Womelsdorf T. A Long-Range Fronto-Parietal 5- to 10-Hz Network Predicts ‘Top-Down’ Controlled Guidance in a Task-Switch Paradigm. Cereb. Cortex bht050 (2013). doi: 10.1093/cercor/bht050 PMC4089379 23448872

[b28] Michel C. M. Electrical Neuroimaging. (Cambridge University Press, 2009).

[b29] Granger C. W. J. Investigating Causal Relations by Econometric Models and Cross-Spectral Methods. Econometrica 37, 424–38 (1969).

[b30] Bressler S. L. & Seth A. K. Wiener–Granger Causality: A well established methodology. NeuroImage 58, 323–329 (2011). 20202481 10.1016/j.neuroimage.2010.02.059

[b31] Astolfi L. *et al.* Comparison of different cortical connectivity estimators for high-resolution EEG recordings. Human Brain Mapping 28, 143–157 (2007). 16761264 10.1002/hbm.20263 PMC6871398

[b32] Baccalá L. A. & Sameshima K. Partial directed coherence: a new concept in neural structure determination. Biol Cybern 84, 463–474 (2001). 11417058 10.1007/PL00007990

[b33] Kamiński M., Ding M., Truccolo W. A. & Bressler S. L. Evaluating causal relations in neural systems: Granger causality, directed transfer function and statistical assessment of significance. Biol Cybern 85, 145–157 (2001). 11508777 10.1007/s004220000235

[b34] Kus R., Kaminski M. & Blinowska K. J. Determination of EEG activity propagation: pair-wise versus multichannel estimate. IEEE Transactions on Biomedical Engineering 51, 1501–1510 (2004). 15376498 10.1109/TBME.2004.827929

[b35] Kamiński M., Blinowska K. & Szelenberger W. Topographic analysis of coherence and propagation of EEG activity during sleep and wakefulness. Electroencephalography and Clinical Neurophysiology 102, 216–227 (1997). 9129577 10.1016/s0013-4694(96)95721-5

[b36] Babiloni F. *et al.* Estimation of the cortical functional connectivity with the multimodal integration of high-resolution EEG and fMRI data by directed transfer function. NeuroImage 24, 118–131 (2005). 15588603 10.1016/j.neuroimage.2004.09.036

[b37] Supp G. G., Schlögl A., Trujillo-Barreto N., Müller M. M. & Gruber T. Directed Cortical Information Flow during Human Object Recognition: Analyzing Induced EEG Gamma-Band Responses in Brain’s Source Space. PLoS ONE 2, e684 (2007). 17668062 10.1371/journal.pone.0000684 PMC1925146

[b38] Gómez-Herrero G., Atienza M., Egiazarian K. & Cantero J. L. Measuring directional coupling between EEG sources. NeuroImage 43, 497–508 (2008). 18707006 10.1016/j.neuroimage.2008.07.032

[b39] Plomp G., Quairiaux C., Michel C. M. & Astolfi L. The physiological plausibility of time-varying Granger-causal modeling: Normalization and weighting by spectral power. NeuroImage 97, 206–216 (2014). 24736179 10.1016/j.neuroimage.2014.04.016

[b40] Brunet D., Murray M. M. & Michel C. M. Spatiotemporal Analysis of Multichannel EEG: CARTOOL. Intell. Neuroscience 2011, 2 1–2 15 (2011). 10.1155/2011/813870 PMC3022183 21253358

[b41] Birot G. *et al.* Head model and electrical source imaging: A study of 38 epileptic patients. NeuroImage: Clinical 5, 77–83 (2014). 25003030 10.1016/j.nicl.2014.06.005 PMC4081973

[b42] Itier R. J. & Taylor M. J. Source analysis of the N170 to faces and objects. Neuroreport 15, 1261 (2004). 15167545 10.1097/01.wnr.0000127827.73576.d8

[b43] Büchel C. & Friston K. J. Modulation of connectivity in visual pathways by attention: cortical interactions evaluated with structural equation modelling and fMRI. Cereb. Cortex 7, 768–778 (1997). 9408041 10.1093/cercor/7.8.768

[b44] McIntosh A. R. *et al.* Network analysis of cortical visual pathways mapped with PET. J. Neurosci. 14, 655–666 (1994). 8301356 10.1523/JNEUROSCI.14-02-00655.1994 PMC6576802

[b45] Saalmann Y. B., Pigarev I. N. & Vidyasagar T. R. Neural Mechanisms of Visual Attention: How Top-Down Feedback Highlights Relevant Locations. Science 316, 1612–1615 (2007). 17569863 10.1126/science.1139140

[b46] Wen X., Yao L., Liu Y. & Ding M. Causal Interactions in Attention Networks Predict Behavioral Performance. J. Neurosci. 32, 1284–1292 (2012). 22279213 10.1523/JNEUROSCI.2817-11.2012 PMC6796284

[b47] Klimesch W., Doppelmayr M., Russegger H., Pachinger T. & Schwaiger J. Induced alpha band power changes in the human EEG and attention. Neuroscience Letters 244, 73–76 (1998). 9572588 10.1016/s0304-3940(98)00122-0

[b48] Lopes da Silva F. Neural mechanisms underlying brain waves: from neural membranes to networks. Electroencephalography and Clinical Neurophysiology 79, 81–93 (1991). 1713832 10.1016/0013-4694(91)90044-5

[b49] Hipp J. F., Engel A. K. & Siegel M. Oscillatory Synchronization in Large-Scale Cortical Networks Predicts Perception. Neuron 69, 387–396 (2011). 21262474 10.1016/j.neuron.2010.12.027

[b50] Schmiedt J. T. *et al.* Beta Oscillation Dynamics in Extrastriate Cortex after Removal of Primary Visual Cortex. J. Neurosci. 34, 11857–11864 (2014). 25164679 10.1523/JNEUROSCI.0509-14.2014 PMC4145181

[b51] Bastos A. M. *et al.* Visual Areas Exert Feedforward and Feedback Influences through Distinct Frequency Channels. Neuron 85, 390–401 (2015). 25556836 10.1016/j.neuron.2014.12.018

[b52] Kerkoerle T. van *et al.* Alpha and gamma oscillations characterize feedback and feedforward processing in monkey visual cortex. PNAS 201402773 (2014). doi: 10.1073/pnas.1402773111 PMC4210002 25205811

[b53] Schmolesky M. T. *et al.* Signal Timing Across the Macaque Visual System. Journal of Neurophysiology 79, 3272–3278 (1998). 9636126 10.1152/jn.1998.79.6.3272

[b54] Nassi J. J. & Callaway E. M. Parallel processing strategies of the primate visual system. Nat Rev Neurosci 10, 360–372 (2009). 19352403 10.1038/nrn2619 PMC2771435

[b55] Maruyama M., Palomo D. D. & Ioannides A. A. Stimulus-contrast-induced biases in activation order reveal interaction between V1/V2 and human MT + . Hum. Brain Mapp. 30, 147–162 (2009). 18041740 10.1002/hbm.20495 PMC6871079

[b56] Sincich L. C., Park K. F., Wohlgemuth M. J. & Horton J. C. Bypassing V1: a direct geniculate input to area MT. Nat Neurosci 7, 1123–1128 (2004). 15378066 10.1038/nn1318

[b57] Rees G. The anatomy of blindsight. Brain 131, 1414–1415 (2008). 18522958 10.1093/brain/awn089 PMC2602754

[b58] Schmid M. C. *et al.* Blindsight depends on the lateral geniculate nucleus. Nature 466, 373–377 (2010). 20574422 10.1038/nature09179 PMC2904843

[b59] Pascual-Leone A. & Walsh V. Fast Backprojections from the Motion to the Primary Visual Area Necessary for Visual Awareness. Science 292, 510–512 (2001). 11313497 10.1126/science.1057099

[b60] Hupé J. M. *et al.* Cortical feedback improves discrimination between figure and background by V1, V2 and V3 neurons. Nature 394, 784–787 (1998). 9723617 10.1038/29537

[b61] Bullier J., Hupé J. M., James A. C. & Girard P. The role of feedback connections in shaping the responses of visual cortical neurons. Prog. Brain Res. 134, 193–204 (2001). 11702544 10.1016/s0079-6123(01)34014-1

[b62] O’Reilly R. C., Wyatte D., Herd S., Mingus B. & Jilk D. J. Recurrent Processing during Object Recognition. Front Psychol 4, 124 (2013). 23554596 10.3389/fpsyg.2013.00124 PMC3612699

[b63] Bullier J. Feedback connections and conscious vision. Trends in Cognitive Sciences 5, 369–370 (2001). 11520692 10.1016/s1364-6613(00)01730-7

[b64] Kennedy H., Dehay C. & Bullier J. Organization of the callosal connections of visual areas v1 and v2 in the macaque monkey. J. Comp. Neurol. 247, 398–415 (1986). 3088065 10.1002/cne.902470309

[b65] Rihs T. A., Michel C. M. & Thut G. Mechanisms of selective inhibition in visual spatial attention are indexed by α-band EEG synchronization. European Journal of Neuroscience 25, 603–610 (2007). 17284203 10.1111/j.1460-9568.2007.05278.x

[b66] Blatt G. J., Andersen R. A. & Stoner G. R. Visual receptive field organization and cortico-cortical connections of the lateral intraparietal area (area LIP) in the macaque. J. Comp. Neurol. 299, 421–445 (1990). 2243159 10.1002/cne.902990404

[b67] Bach M. The Freiburg Visual Acuity test–automatic measurement of visual acuity. Optom Vis Sci 73, 49–53 (1996). 8867682 10.1097/00006324-199601000-00008

[b68] Rossion B. & Pourtois G. Revisiting Snodgrass and Vanderwart’s object pictorial set: the role of surface detail in basic-level object recognition. Perception 33, 217–236 (2004). 15109163 10.1068/p5117

[b69] Sadr J. & Sinha P. Object recognition and Random Image Structure Evolution. Cognitive Science 28, 259–287 (2004).

[b70] Peirce J. W. PsychoPy—Psychophysics software in Python. Journal of Neuroscience Methods 162, 8–13 (2007). 17254636 10.1016/j.jneumeth.2006.11.017 PMC2018741

[b71] Peirce J. W. Generating Stimuli for Neuroscience Using PsychoPy. Front Neuroinformatics 2, (2009). 10.3389/neuro.11.010.2008 PMC2636899 19198666

[b72] Berman R. A. *et al.* Cortical networks subserving pursuit and saccadic eye movements in humans: an FMRI study. Hum Brain Mapp 8, 209–225 (1999). 10619415 10.1002/(SICI)1097-0193(1999)8:4<209::AID-HBM5>3.0.CO;2-0 PMC6873313

[b73] Perrin F., Pernier J., Bertrand O. & Echallier J. F. Spherical splines for scalp potential and current density mapping. Electroencephalography and Clinical Neurophysiology 72, 184–187 (1989). 2464490 10.1016/0013-4694(89)90180-6

[b74] Seth A. K. A MATLAB toolbox for Granger causal connectivity analysis. Journal of Neuroscience Methods 186, 262–273 (2010). 19961876 10.1016/j.jneumeth.2009.11.020

[b75] Coito A. *et al.* Dynamic directed interictal connectivity in left and right temporal lobe epilepsy. Epilepsia 56, 207–217 (2015). 25599821 10.1111/epi.12904

[b76] Plomp G., Leeuwen C. van & Ioannides A. A. Functional specialization and dynamic resource allocation in visual cortex. Human Brain Mapping 31, 1–13 (2010). 19621367 10.1002/hbm.20840 PMC6871229

[b77] Milde T. *et al.* A new Kalman filter approach for the estimation of high-dimensional time-variant multivariate AR models and its application in analysis of laser-evoked brain potentials. NeuroImage 50, 960–969 (2010). 20060483 10.1016/j.neuroimage.2009.12.110

[b78] Astolfi L. *et al.* Tracking the Time-Varying Cortical Connectivity Patterns by Adaptive Multivariate Estimators. IEEE Transactions on Biomedical Engineering 55, 902–913 (2008). 18334381 10.1109/TBME.2007.905419

[b79] Frei E. *et al.* Localization of MDMA-induced brain activity in healthy volunteers using low resolution brain electromagnetic tomography (LORETA). Human Brain Mapping 14, 152–165 (2001). 11559960 10.1002/hbm.1049 PMC6872058

[b80] Haufe S., Nikulin V. V., Müller K.-R. & Nolte G. A critical assessment of connectivity measures for EEG data: A simulation study. NeuroImage 64, 120–133 (2013). 23006806 10.1016/j.neuroimage.2012.09.036

[b81] Vinck M. *et al.* How to detect the Granger-causal flow direction in the presence of additive noise? NeuroImage doi: 10.1016/j.neuroimage.2014.12.017 (2015) 25514516

[b82] Cohen J. A power primer. Psychol Bull 112, 155–159 (1992). 19565683 10.1037//0033-2909.112.1.155

[b83] Gardner M. J. & Altman D. G. Confidence intervals rather than P values: estimation rather than hypothesis testing. Br Med J (Clin Res Ed) 292, 746–750 (1986). 10.1136/bmj.292.6522.746 PMC1339793 3082422

[b84] He B. *et al.* eConnectome: A MATLAB Toolbox for Mapping and Imaging of Brain Functional Connectivity. J Neurosci Methods 195, 261–269 (2011). 21130115 10.1016/j.jneumeth.2010.11.015 PMC3244474

